# Anti-EGFR monoclonal antibody Cetuximab displays potential anti-cancer activities in feline oral squamous cell carcinoma cell lines

**DOI:** 10.3389/fvets.2022.1040552

**Published:** 2022-11-17

**Authors:** Gennaro Altamura, Giuseppe Borzacchiello

**Affiliations:** General Pathology and Anatomic Pathology Section, Department of Veterinary Medicine and Animal Productions, University of Naples Federico II, Naples, Italy

**Keywords:** cat, Cetuximab, EGFR, Akt, molecular targeted therapy, oral squamous cell carcinoma

## Abstract

Feline oral squamous cell carcinoma (FOSCC) is a malignant tumor characterized by an aggressive behavior and poor prognosis, for which no fully effective therapies are available. Studies of comparative oncology suggest that epidermal growth factor receptor (EGFR) may be a therapeutic target in FOSCC, similarly to human head and neck SCC (HNSCC), where the use of anti-EGFR monoclonal antibody Cetuximab has entered the clinical practice. The aim of this study was to assess the efficacy of Cetuximab in three validated preclinical models of FOSCC (SCCF1, SCCF2, SCCF3). Sequencing of tyrosine kinase domain of EGFR in the cell lines revealed a wild-type genotype, excluding the presence of activating mutations. Western blotting experiments demonstrated that Cetuximab inhibited activation of EGFR and its downstream kinase Akt in SCCF1, SCCF2 and SCCF3 along with HNSCC cell line CAL 27 included as control. Importantly, CCK-8 and trypan blue exclusion assays revealed that treatment with Cetuximab caused a decrease in cell proliferation and cell viability in all cell lines, with a general dose- and time-dependent trend. Cell death induced by Cetuximab was associated with cleavage of PARP, indicating occurrence of apoptosis. Taken together, our data suggest that Cetuximab exerts potential anti-cancer activities in FOSCC, paving the way for future translational studies aimed at assessing its employment in the therapy of this lethal cancer of cats.

## Introduction

Squamous cell carcinoma (SCC) is the most common oral cancer in cats (1). It is characterized by aggressive behavior, frequent local invasion and high metastatic potential (1). Current options of treatment such as surgery, radiation therapy (RT) and chemotherapy (CT) are mostly unsuccessful, so that the disease displays high degree of relapse and poor prognosis (1). Palliative care may be adopted to transiently improve the quality of life, however patients are often subjected to euthanasia due to complications associated with tumor progression (1). Therefore, feline oral SCC (FOSCC) represents a major challenge in veterinary oncology, hence the need to identify new molecular targets with the aim of devising new experimental therapies.

Epidermal growth factor receptor (EGFR) is a membrane tyrosine kinase receptor (TKR) that regulates homeostasis and growth of epithelial cells (2). Once activated by its natural ligand (EGF), EGFR triggers the Ras-mitogen-activated-protein kinases (MAPK) and PI3K-Akt signal transduction pathways resulting in phosphorylation of the terminal kinases ERK and Akt, respectively, which in turn regulate expression of genes involved in cell proliferation and inhibition of apoptosis (3). EGFR pathway is upregulated in several epithelial tumors, therefore being worthy of scientific efforts toward the development of molecular targeted therapies based on the use of TK inhibitors (TKI) and anti-EGFR monoclonal antibodies in human and veterinary medicine (2–6).

Cetuximab is a recombinant, chimeric monoclonal antibody raised against human EGFR: its Fv (variable, antigen binding) region, of murine origin, is able to bind to the N-terminal region of human EGFR and is fused to the Fc of human IgG1 light and heavy chains (3). The affinity of Cetuximab for EGFR is even higher than EGF, therefore it efficiently binds to receptor extracellular domain, resulting in the inhibition of its activation and thus switching-off its downstream signal transduction pathways (3). Cetuximab may exert this function with additional mechanisms, for instance by inducing internalization of EGFR with consequent downregulation of the receptor as well as by antibody dependent cell-mediated cytotoxicity (3, 7). This high specificity and redundancy in functioning modalities has made Cetuximab the ideal compound for targeted therapy in tumors driven by EGFR upregulation, such as human head and neck SCC (HNSCC) (3).

Indeed, near 90% of HNSCC show over-expression of EGFR, in association with a more aggressive disease and lower survival rate; therefore, Cetuximab (marketed as Erbitux^®^) has entered the routine therapeutic protocols in association with CT and RT for patients with locally advanced and recurrent metastatic disease (3, 8). Moreover, it has been considered a leading candidate in the de-escalation strategies aimed at establishing targeted and less invasive treatments, particularly in the sub-group of HNSCC associated with human papillomavirus (HPV) infection (20–25%), given their more favorable outcome (9).

The similarities between human and pet tumors lay the foundations of “comparative oncology,” with the dual aim of developing therapies in humans based on the studies in spontaneous animal models and/or translating the therapies used in humans into veterinary medicine (5). At this regard, FOSCC shares several biological properties with HNSCC such as over-expression of EGFR (10) and, in a subset of tumors, association with PV infection, mainly by *Felis catus* PV type 2 (FcaPV-2), thus it is considered a reliable spontaneous animal model of human counterpart (11–13). Furthermore, several studies suggest that expression of EGFR is a marker of poor prognosis also in FOSCC (10, 14, 15) and, accordingly, that EGFR plays a key role in tumor cells proliferation and survival, suggesting that it may be a promising molecular target in this feline cancer as well (16).

The aim of this study was to investigate the effects of treatment with Cetuximab in three FOSCC cell lines using a human OSCC cell line as a control, by evaluating inhibition of EGFR pathway, impairment of cell proliferation and cell viability, in order to obtain a preliminary assessment of its potential as a possible future candidate for the treatment of this tumor.

## Materials and methods

### Cell lines and treatments conditions

FOSCC cell lines SCCF1 (laryngeal SCC), SCCF2 (gingival SCC) and SCCF3 (tongue SCC), developed in the Rosol laboratory, were a kind gift from Professor T.J. Rosol (The Ohio State University) and have been cultured as described elsewhere (17–19). Cetuximab-sensitive cell line CAL 27 (human tongue SCC) was purchased at ATCC cell bank (#CRL-2095 ATCC, Manassas, VA, USA) and cultured according to product datasheet (20). For evaluation of biochemical effects of Cetuximab treatment on EGFR pathway, CAL 27 and SCCF3 were seeded in 6-well plates at 1 × 10^5^ density and, after 24 h, treated at 100 μg/mL for 3, 6 and 12 h, to be subjected to Western blotting as described below. Experimental conditions for evaluation of cell growth inhibition were calibrated by treating SCCF2 cell line with Cetuximab (#A2000 Selleckchem, Planegg, Germany) along with CAL 27. Cells were incubated with increasing doses of Cetuximab (10, 50, 100 μg/mL) diluted in culture medium for 24, 48, and 72 h. In control plates, Cetuximab was replaced with phosphate buffered saline (PBS). Relative growth inhibition in treated *vs*. control plates was calculated as previously described (21).

### Cell counting kit 8 (CCK-8) assay

Cells were seeded at 5 × 10^3^ density in 96-well plates and, after 24 h, incubated with Cetuximab at 50 and 100 μg/mL. CCK-8 assay (#ab228554 Abcam, Cambridge, UK) was performed to assess cell proliferation according to manufacturer protocol and absorbance at 460 nm was measured after 24, 48 and 72 h as reported elsewhere (22). Each treatment was performed in triplicate and repeated twice. Decrease in absorbance at each dose was expressed as percentage compared with the respective control plate treated with PBS.

### Trypan blue exclusion assay

Cells were treated with Cetuximab at 50 and 100 μg/mL, harvested after 48 and 72 h and subjected to trypan blue exclusion assay to assess cell viability as previously described (21). Changes in cell viability at each treatment condition were calculated and expressed as percentage compared with the respective control plate treated with PBS.

### Western blotting and antibodies

Cells were harvested by trypsinization and subjected to protein extraction, protein quantification, sodium dodecyl sulfate- polyacrylamide gel electrophoresis, Western blotting (WB) and protein bands detection as previously described (21). The following antibodies were employed at 1:1,000 dilution: EGFR (#MS-378-P0, Thermo Fisher Scientific), phospho-EGFR (pEGFR, #3777 Cell Signaling Technology, Danvers, MA, USA), ERK (# 4695 Cell Signaling Technology, Danvers, MA, USA), phospho-ERK (pERK, #4370 Cell Signaling Technology, Danvers, MA, USA), Akt (#2920 Cell Signaling Technology, Danvers, MA, USA), phospho-Akt (pAkt, #4060 Cell Signaling Technology, Danvers, MA, USA), PARP (#9542 Cell Signaling Technology, Danvers, MA, USA), β-actin (#4967 Cell Signaling Technology, Danvers, MA, USA), BAX (#sc-493 Santa Cruz Biotechnology, Santa Cruz, CA, USA). Information regarding antibodies clones and reactivity in cat are available in Supplementary Table 1. Further validation of antibodies was guaranteed by running feline samples along with CAL 27 protein extracts.

### Sequencing of feline EGFR TK region and alignment of feline and human EGFR protein

Reverse transcription PCR (RT-PCR) and sequence analysis (BMR Genomics, Padova, Italy) of feline EGFR regions spanning the TK domain (TKD) in SCCF2 and SCCF3 were performed by using sequencing strategy, PCR protocol, and the following primers sets A: for 5′GGA GAA GCT CCC AAC CAG GCT3′, rev 5′GAT AGG CAC TTT GCC TCC TTC3′, B: for 5′GAA TAT CAC CTG CAC AGG AC3′, rev 5′GCC ATC ACG TAA GCT TCA TC3′ and C: for 5′TGC GAA GGG CAT GAA CTA C3′, rev 5′ACT CAT CGG CAT CTA CGA C3′ as reported elsewhere for SCCF1 (16). Alignment of feline (Genbank: ALJ56200.1) to human EGFR protein (AIC61960.1) was performed by using NCBI/Blastp tool.

### Statistical analysis

For statistical analysis, paired Student t-test was run to analyze the differences between each treatment *vs*. control, ANOVA was performed to investigate the differences between the mean of three groups of samples. Statistical tests were performed using SPSS Statistics for Windows, version 17.1 (SPSS Inc. Released 2008, Chicago, IL, USA).

## Results

### Sequencing of EGFR TKD in SCCF2 and SCCF3 cell lines and alignment of feline and human EGFR protein

One of the mechanisms leading to EGFR dysregulation in cancer is the occurrence of activating mutations in the TKD (2), hence we performed sequence analysis of a region of 1329 bp encompassing this domain (~34% of EGFR transcript) as the preliminary step of our experimental plan. Sequencing of EGFR in SCCF1 had yielded a wild-type sequence (Genbank: HQ185236) for TKD in a previous study (16) and here, the sequences obtained for SCCF2 and SCCF3 by using the analogous RT-PCR approach were identical to that of SCCF1. Sequence data were deposited in Genbank with the accession numbers OP643788 and OP688567. Additionally, alignment of feline EGFR to human protein produced 89% sequence identity, increasing to 92% when considering conservative substitutions (not shown). Most importantly, when aligning the regions encompassing the EGFR domain responsible for Cetuximab binding (domain III) (23) (Supplementary Figure 1), the percentages of homology grew to 91 and 96%, respectively, strengthening the rationale for the following experimental steps.

### Cetuximab inhibits EGFR pathway in FOSCC cell lines

**Figure 1:**
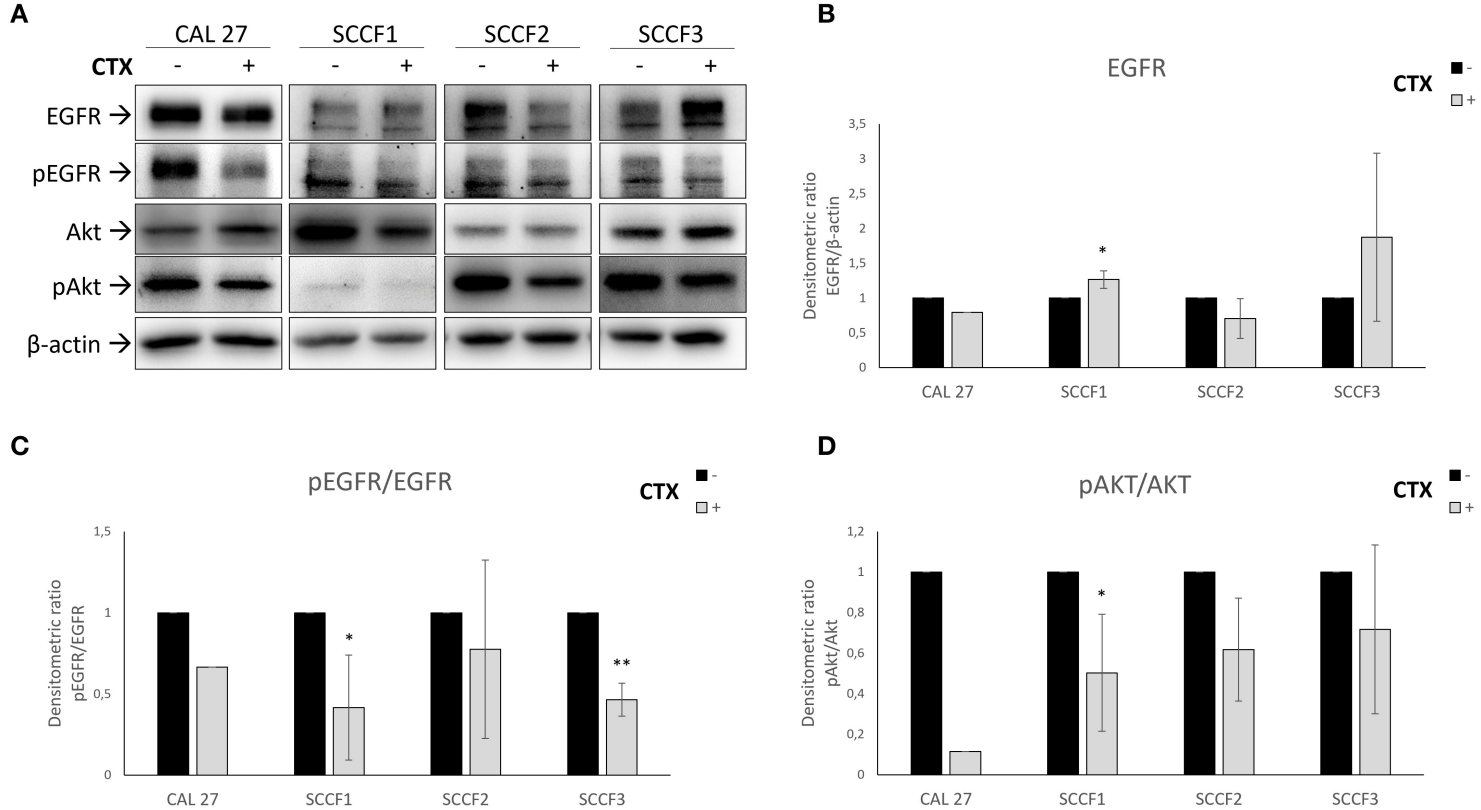
Inhibition of EGFR pathway by Cetuximab in feline oral squamous cell carcinoma cell lines (SCCF1, SCCF2, SCCF3). **(A)** Cells were incubated with Cetuximab (CTX) at 100 μg/mL for 12 h and analyzed by western blotting (WB) for EGFR, phospho-EGFR (pEGFR), Akt and phospho-Akt (pAkt). Cetuximab-sensitive CAL 27 cells were included as control. The treatment (+) induced reduction of pEGFR levels in all cell lines with respect to untreated control (–), along with decreased EGFR in SCCF2 and its accumulation in SCCF3 and SCCF1. Impairment of EGFR by Cetuximab was accompanied by a decrease of pAkt levels. WB for β-actin antibody ensured comparable protein loading and allowed normalization. **(B)** Densitometric analysis of EGFR expressed as densitometric ratio with β-actin. **(C,D)** Densitometric analysis of pEGFR and pAkt expressed as densitometric ratio pEGFR/EGFR and pAkt/Akt, respectively. Standard deviations are from three repeated, independent experiments (*: statistically significant by *t*-test, *P* ≤ 0.05; **: statistically significant by *t*-test, *P* ≤ 0.01).

To evaluate the biochemical effects of Cetuximab in FOSCC, a preliminary experiment was performed in SCCF3, along with Cetuximab-sensitive CAL 27 cells: WB for EGFR and its activated form pEGFR demonstrated a time-dependent inhibition of receptor phosphorylation upon treatment at 100 μg/mL for 3, 6 and 12 h, therefore this latter incubation time was chosen to carry out the analysis in all feline cell lines (see Supplementary Figure 2). Expression and activation of EGFR was confirmed in SCCF1, SCCF2 and SCCF3 and, notably, incubation with Cetuximab caused a decrease of pEGFR in treated cells when compared with their respective untreated counterpart, including in CAL 27 employed as control (Figure 1A). Interestingly, the drug also caused a downregulation of total EGFR in SCCF2, whilst an increase could be observed in treated SCCF3 and, although slightly, in SCCF1 (Figure 1A). Densitometric analysis of EGFR and pEGFR/EGFR ratio confirmed WB results (Figures 1B,C). To further investigate the effects on downstream PI3K-Akt pathway, WB for Akt and its activated form pAkt was performed: Akt was expressed and activated at control conditions and, consistently with the data above, EGFR impairment was accompanied by decreased pAkt levels in all treated cells (Figure 1A), as confirmed by densitometric analysis of pAkt/Akt ratio (Figure 1D). The MAPK downstream kinase ERK was expressed and phosphorylated at steady state in all cell lines and, whilst Cetuximab caused the expected decrease in pERK levels in CAL 27, data from treatments of feline cells appeared inconclusive, given that in most of the experiments a surprising increase was observed *via* WB (see Supplementary Figure 3).

### Cetuximab impairs cell proliferation in FOSCC cell lines

Once the biochemical activity of Cetuximab had been demonstrated, we aimed at investigating its impact on cell proliferation. In tritration experiments, the doses at 50 and 100 μg/mL had caused a relative growth inhibition in a dose-dependent manner in CAL 27 at each of time points (24, 48, 72 h) and in SCCF2 at 48 and 72 h (see Supplementary Figure 4), therefore these two concentrations were selected to perform cell proliferation tests in feline cells by CCK-8 assay.

**Figure 2:**
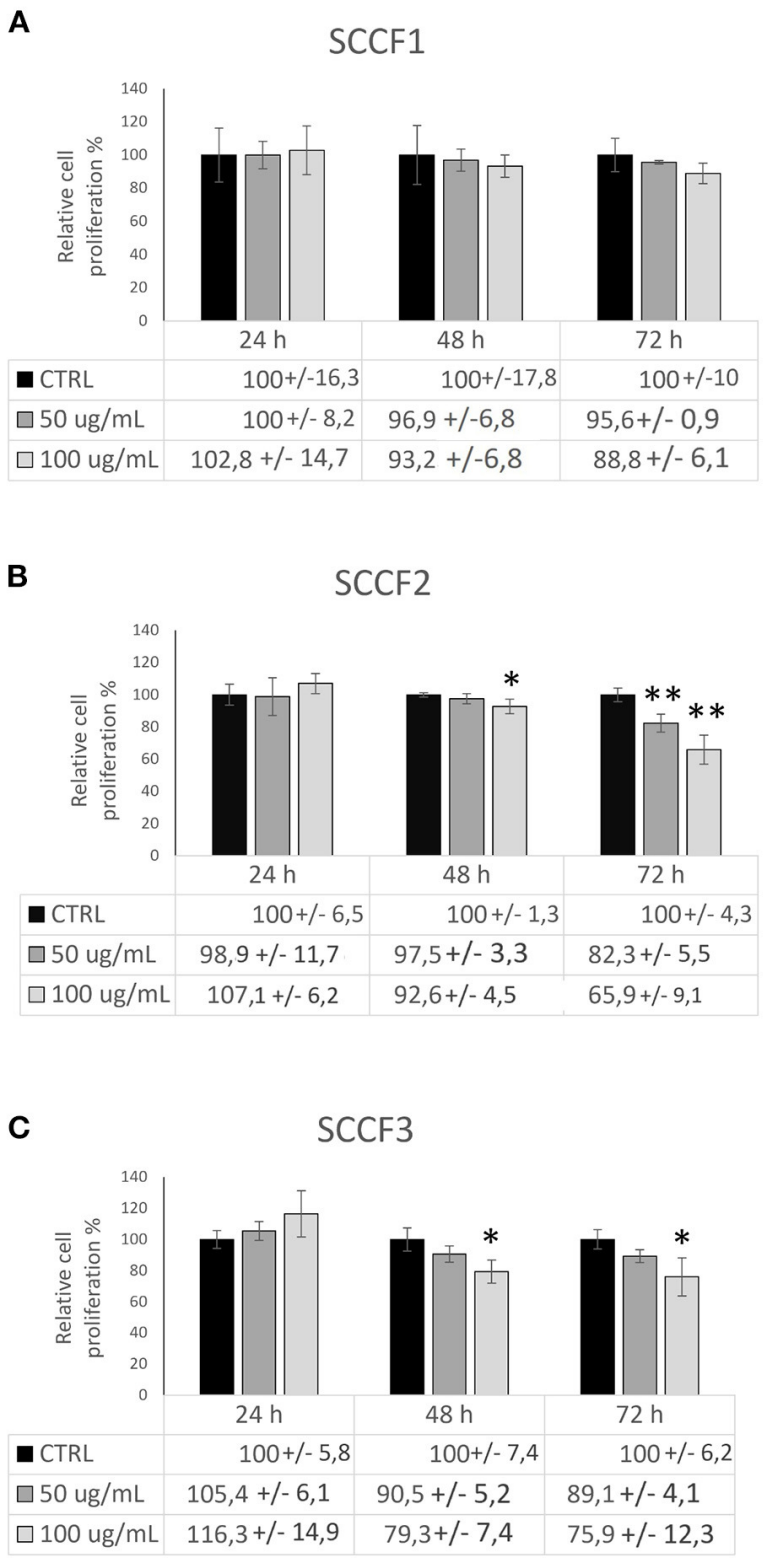
Impact of Cetuximab on cell proliferation in feline oral squamous cell carcinoma cell lines. Cells treated with Cetuximab at 50 and 100 μg/mL were analyzed by CCK-8 assay after 24, 48, and 72 h. The treatment induced a dose- and time-dependent impairment of cell proliferation in SCCF1 **(A)**, SCCF2 **(B)** and SCCF3 **(C)**. Data are presented as percentage (%) compared with the respective control (CTRL) and represent mean ± SD from three replicates. Paired Student's *t*-tests were used to compare each treatment to control (*: statistically significant by *t*-test, *P* ≤ 0.05; **: statistically significant by *t*-test, *P* ≤ 0.01). ANOVA analysis showed also statistically significant difference between all group means at 72 h in SCCF2 (F = 34.7, *P* < 0.01), 48 (F = 8.644, *P* < 0.05) and 72 h (F = 9.376, *P* < 0.01) in SCCF3.

In accordance with the calibration experiment, no effect on cell proliferation was detected at 24 h of treatment neither in SCCF2 nor in the other FOSCC cell lines (Figure 2). In SCCF1 cells, Cetuximab caused a slight, not statistically significant decrease of proliferative abilities in a dose-dependent manner after 72 h, although a similar behavior could be already seen at 48 h (Figure 2A). In SCCF2 cell line, 48 h were sufficient to appreciate a reduction of cell proliferation upon incubation with Cetuximab in a dose-dependent manner, which became more glaring after 72 h (Figure 2B). SCCF3 appeared to be sensitive to earlier time of treatments, given that a noticeable dose-dependent effect of Cetuximab on cell proliferation was appreciable already at 48 h, to become slightly more evident at 72 h of treatment (Figure 2C). Treatment of control cells CAL 27 at 100 μg/mL for 48 h caused a decrease of cell proliferation with a % change comparable to that obtained in feline cells, strengthening the reliability of the procedure and the obtained results (Supplementary Figure 5A).

### Cetuximab decreases cell viability *via* apoptosis in FOSCC cell lines

**Figure 3:**
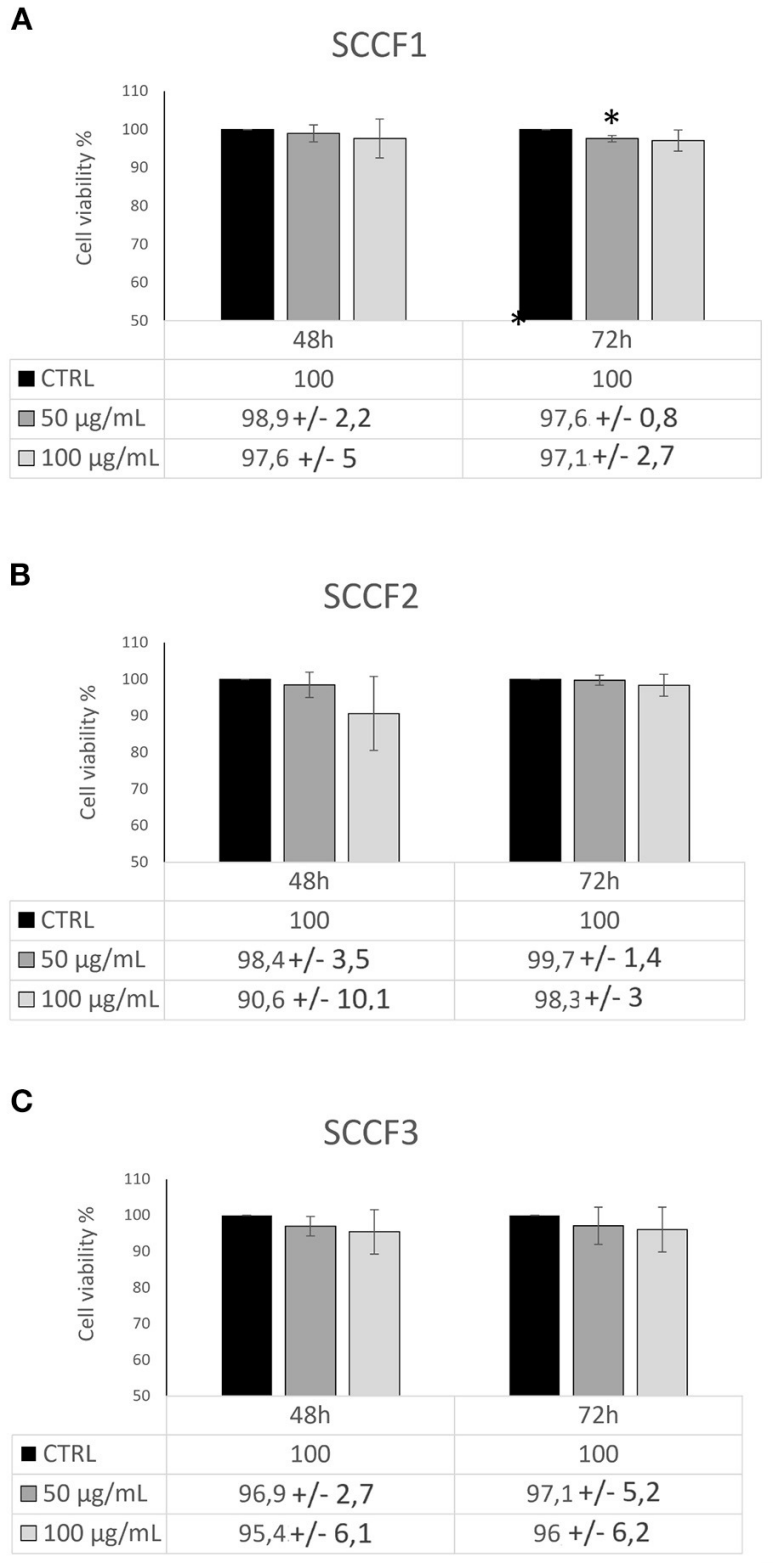
Impact of Cetuximab on cell viability in feline oral squamous cell carcinoma cell lines. Cells treated with Cetuximab at 50 and 100 μg/mL were analyzed by trypan blue exlclusion assay after 48 and 72 h. The treatment induced decrease in cell viability in SCCF1 **(A)**, SCCF2 **(B)** and SCCF3 **(C)**. Data are presented as percentage (%) compared with the respective control (CTRL) and represent mean ± SD from three independent experiments. Paired Student's *t*-tests were used to compare treatments to control; differences were not statistically significant, except were indicated (*P ≤ 0.05).

**Figure 4:**
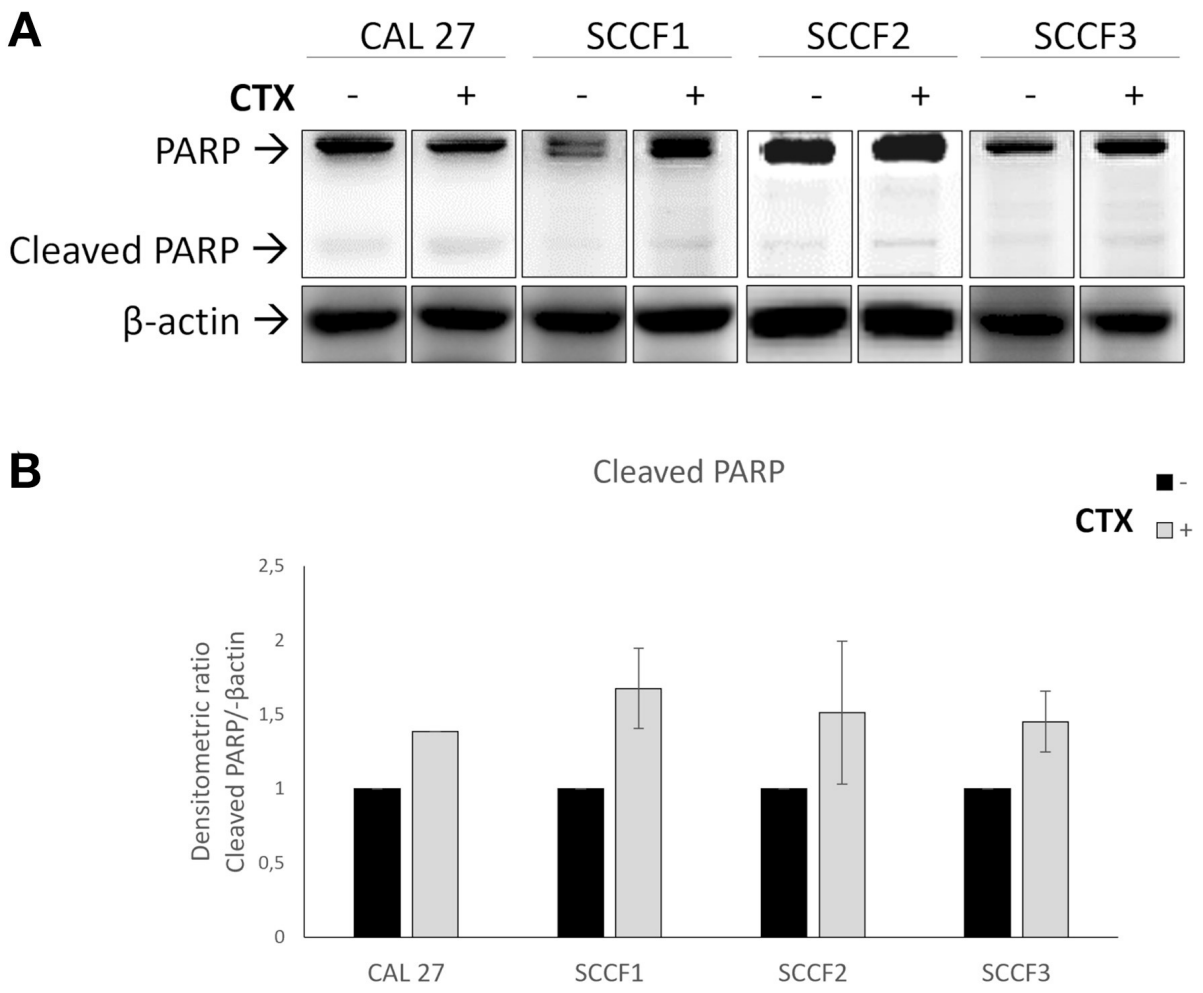
Cleavage of the proapoptotic marker PARP in feline oral squamous cell carcinoma cell lines (SCCF1, SCCF2, SCCF3) treated with Cetuximab. **(A)** Cells treated with Cetuximab (CTX) at 100 μg/mL were analyzed by western blotting (WB) for PARP after 48/72 h. Cetuximab-sensitive CAL 27 cells were included as control. The treatment (+) induced an increase of cleaved PARP in all cell lines compared to untreated control (–). WB for β-actin antibody ensured comparable protein loading and allowed normalization. Paired boxes for each cell line are cut from the same membrane at the same exposure time and properly aligned according to the molecular marker loaded onto the gel. Full scans from original gels are shown in Supplementary Figure 8. **(B)** Densitometric analysis of cleaved PARP expressed as densitometric ratio with β-actin. Standard deviations are from two repeated, independent experiments yielding comparable results.

To further assess whether Cetuximab would cause cell death other than impaired cell growth, trypan blue exclusion assays were conducted. The obtained results revealed that treatment with Cetuximab was associated with a slight and often not statistically significant decrease of cell viability in feline cells (Figure 3), however the reliability of methodology and results was guaranteed by the fact that similar faint effects were obtained in control cells CAL 27, in agreement with previous studies (24, 25) (Supplementary Figure 5B). More in detail, treatment of SCCF1 slightly reduced cell viability in a dose-dependent manner at 72 h, despite a similar behavior was observable already at 48 h (Figure 3A). In SCCF2 cell line, reduction of cell viability was more evident at 48 h, still with a modality related to increasing concentrations of Cetuximab (Figure 3B), whilst in SCCF3 the treatment induced a mild decrease of viable cells with a dose-dependent manner after 48 h, that was maintained at similar levels at 72 h (Figure 3C). To gain insights into what cell death process the observed decrease in cell viability was associated to, WB for apoptotic marker PARP (Figure 4A) followed by densitometric analysis (Figure 4B) was performed. Results showed a mild increase in PARP cleavage upon treatment with Cetuximab in all cell lines as well as in CAL 27 indicating that, despite weak, the cytotoxic effect was associated with apoptosis. Occurrence of apoptosis was further confirmed by WB showing increase of the apoptotic marker BAX in treated SCCF2 (Supplementary Figure 6).

## Discussion

The anti-EGFR monoclonal antibody Cetuximab has been approved for the treatment of HNSCC (3). Previous studies *in vivo* (14, 15) and *in vitro* (16, 26, 27) suggested that EGFR might be a therapeutic target also in FOSCC, prompting us to test the anti-cancer potential of Cetuximab in well-characterized cell lines derived from this tumor (17–19).

Activating mutations in the TKD of EGFR gene may be leading events in tumor development (2). Sequencing data obtained here for SCCF2 and SCCF3, along with those reported for SCCF1 in a previous study, revealed a wild-type genotype, suggesting that EGFR dysregulation in FOSCC is not driven by TKD mutations (16). Importantly, the presence of these latter is suspected to confer insensitivity to Cetuximab and has been associated with poor response in treated HNSCC patients (28, 29), therefore the lack of any TKD mutation in FOSCC cell lines heralded a potential response to treatment. Additionally, Cetuximab binding domain (domain III) (23) appeared highly conserved in feline EGFR, further strengthening the rationale of this study.

Our WB data, guaranteed by the use of antibodies already validated in cat, confirmed expression and activation of EGFR and ERK in all of the three cell lines in agreement with previous work (21, 27, 30), and provided first evidence of Akt activation at the steady state in SCCF1 and SCCF2, implying a possible role of these molecules in FOSCC pathogenesis. It has been previously shown that SCCF2 and SCCF3 harbor transcriptionally active FcaPV-2 (31) and that the viral oncoprotein E6 enhances phosphorylation of ERK and Akt but not EGFR (32), therefore their basal activation in these two cell lines might be related in part to events driven by viral oncogenesis. Additionally, EGFR/ERK pathway had been described to be activated by extra-telomeric functions of telomerase reverse transcriptase in SCCF1/2/3, further explaining our data (21). Most importantly, treatment with Cetuximab resulted in a decrease of pEGFR levels in all cell lines, consistently with its canonical function relying in the inhibition of EGF binding and thus of receptor phosphorylation (3, 7). Generally, TKR activation triggers endocytosis followed by lysosomal degradation of ligand-receptor complex (3, 7), therefore the EGFR increase observed in SCCF3 and SCCF1 treated with Cetuximab may be suggestive of an accumulation of inactive receptor subsequent to its reduced internalization; in veterinary experimental oncology, similar phenomena have been observed for PDGFβR in equine sarcoid derived cell lines treated with the TKI imatinib mesylate, strengthening this hypothesis (22). Of note, additional mechanisms of action have been described for Cetuximab, among these the fostering of antibody-dependent receptor internalization and degradation (3, 7), and this may explain the downregulation of total EGFR observed in treated SCCF2.

Regardless of the possible mechanism, inhibition of EGFR by Cetuximab in feline cells was accompanied by an expected decrease of the downstream effector pAkt but not pERK, for which even an unexpected increase could be observed. Previous studies report similar findings in OSCC cell lines and describe this phenomenon as an adaptive ERK signaling in response to treatment with Cetuximab as well as other CT agents, which however is not sufficient to completely counteract the functionality of these drugs, in agreement with our data (33, 34); combination of Cetuximab plus MAPK inhibitors has been shown to exert an enhanced, synergic anti-cancer effect on treated cells, and this could be a prospect for our future lines of research (33). Another possible explanation to our results might rely in the fact that, despite it is generally considered a pro-survival factor, ERK may also exert some pro-apoptotic functions and confer sensitivity to anti-cancer treatments (35, 36).

Most importantly, the treatment induced an impairment of cell proliferation in feline cells comparable to control cells CAL 27, being suggestive of potential therapeutic applications in FOSCC *in vivo* as in human counterpart. In addition, we observed an impairment of cell viability in treated cells, albeit very slight, in agreement with literature indicating that the biological effects of Cetuximab in human cells are mainly related to its cytostatic properties and less to induction of cell death (37, 38). Nevertheless, previous studies suggest that cell death induced by Cetuximab may be related to apoptosis associated with PARP cleavage (39, 40) and BAX accumulation (38), in accordance with our WB data.

Recent advances in comparative oncology have incentivized the adaptation of small molecule inhibitors and monoclonal antibodies from human to veterinary medicine, hence the rationale for this study (4). However, the use of Cetuximab in cat may present some complications: its human molecular backbone (3) may trigger an anti-antibody response by the host immune system, leading to severe side-effects and decline of treatment efficiency (6, 41). Nevertheless, studies in canine mammary cancer demonstrate that “speciation” of Cetuximab Fc is feasible, with the antibody preserving its affinity for EGFR and its anti-cancer effects in tumor cell lines, thus overcoming this issue (5, 41); therefore, in light of our protein alignment data and considering the high homology between canine, feline and human EGFR genes (5, 26), a similar future scenario is conceivable also in cat.

In conclusion, this study demonstrates that Cetuximab exerts potential anti-cancer activities in FOSCC cell lines and paves the way for translational studies aimed at bridging the gap between benchside and bedside, in order to develop new therapies for this lethal cancer of cats.

## Data availability statement

The sequence data presented in the study are deposited in Genbank (https://www.ncbi.nlm.nih.gov/genbank/), accession numbers OP643788 and OP688567.

## Author contributions

GA performed the experiments. GA and GB conceived the study and drafted the manuscript. All authors contributed to the article and approved the submitted version.

## Funding

This work was financially supported by a grant from the Board of Directors, University of Naples Federico II.

## Conflict of interest

The authors declare that the research was conducted in the absence of any commercial or financial relationships that could be construed as a potential conflict of interest.

## Publisher's note

All claims expressed in this article are solely those of the authors and do not necessarily represent those of their affiliated organizations, or those of the publisher, the editors and the reviewers. Any product that may be evaluated in this article, or claim that may be made by its manufacturer, is not guaranteed or endorsed by the publisher.
