# Supplementary material for: Anti-EGFR monoclonal antibody Cetuximab displays potential anti-cancer activities in feline oral squamous cell carcinoma cell lines
Source: Front Vet Sci. 2022 Nov 17;9:1040552. doi: 10.3389/fvets.2022.1040552 (PMC9712204; doi:10.3389/fvets.2022.1040552)
Supplement: Supplementary file 1 [file Data_Sheet_1.PDF]

Supplementary Material

Table 1

Antibody	Manufacturer	Catalog #	Clone	Reactivity On Cat
EGFR	ThermoFisher Scientific	MS-378-P0	111.6	Altamura et al., 2020 (1)
pEGFR	Cell Signaling	3777	D7A5	*
Akt	Cell Signaling	2920	40D4	*
pAkt	Cell Signaling	4060	D9E	Sanz Ressel et al., 2021 (2)
ERK	Cell Signaling	4695	137F5	Bergkvist et al., 2011 (3)
pERK	Cell Signaling	4370	D13.14.4E	Bergkvist et al., 2011 (3)
PARP	Cell Signaling	9542	Polyclonal	Altamura et al., 2014 (4)
BAX	Santa Cruz	sc-493	Polyclonal	Altamura et al., 2013 (5)

Table 1. Primary antibodies used for Western blotting analysis. For each antibody, the manufacturer, the catalog number (#), the clone and the reference ensuring reactivity on cat are listed (*not available, the reactivity in feline cells was ensured by Western blotting along with CAL 27 as antibody control).

1. Altamura G, Degli Uberti B, Galiero G, De Luca G, Power K, Licenziato L, et al. The Small Molecule Bibr1532 Exerts Potential Anti-Cancer Activities in Preclinical Models of Feline Oral Squamous Cell Carcinoma through Inhibition of Telomerase Activity and Down-Regulation of Tert. Front Vet Sci (2020) 7:620776. doi: 10.3389/fvets.2020.620776.

2. Sanz Ressel BL, Massone AR, Barbeito CG. Persistent Activation of the Mammalian Target of Rapamycin Signalling Pathway in Cutaneous Squamous Cell Carcinomas in Cats. Vet Dermatol (2021) 32(6):675-e180. doi: 10.1111/vde.13001

3. Bergkvist GT, Argyle DJ, Pang LY, Muirhead R, Yool DA. Studies on the Inhibition of Feline Egfr in Squamous Cell Carcinoma: Enhancement of Radiosensitivity and Rescue of Resistance to Small Molecule Inhibitors. Cancer Biol Ther (2011) 11(11):927-37. doi: 10.4161/cbt.11.11.15525.

4. Altamura G, Corteggio A, Accardi R, Tommasino M, Conte A, Borzacchiello G. Felis catus papillomavirus type-2 E6 inhibits UVB-induced apoptosis in feline epithelial cells. In: Tommasino M, editor. Proceedings of the 3rd Meeting on Emerging Oncogenic Viruses; 2014 Giu 4-8. p. 107. http://hdl.handle.net/11588/588199

5. Altamura G, Corteggio A, Pacini L, Conte A, Pierantoni GM, Tommasino M, et al. Transforming Properties of Felis Catus Papillomavirus Type 2 E6 and E7 Putative Oncogenes in Vitro and Their Transcriptional Activity in Feline Squamous Cell Carcinoma in Vivo. Virology (2016) 496:1-8. doi: 10.1016/j.virol.2016.05.017

Supplementary Figure 1

Download | Graphics

unnamed protein product

Sequence ID: **Query_415073** Length: **286** Number of Matches: **1**

Range 1: 1 to 286 Graphics

▼ Next Match ▲ Previous Match

Score	Expect	Method	Identities	Positives	Gaps
546 bits(1407)	0.0	Compositional matrix adjust.	260/286(91%)	275/286(96%)	0/286(0%)

```
Feline → Query  1    YSFGATCVKKCPRNYVVTDHGSCVRACSSDSYEVEEDGVRKCKKCEGPCRKVCNGIGIGE  60
                     YSFGATCVKKCPRNYVVTDHGSCVRAC +DSYE+EEDGVRKCKKCEGPCRKVCNGIGIGE
Human → Sbjct  1    YSFGATCVKKCPRNYVVTDHGSCVRACGADSYEMEEDGVRKCKKCEGPCRKVCNGIGIGE  60

        Query  61   FKDTLSINATNIKHFKNCTSISGDLHILPVAFRGDSFTHTPPLDPKELDILKTVKEITGF  120
                     FKD+LSINATNIKHFKNCTSISGDLHILPVAFRGDSFTHTPPLDP+ELDILKTVKEITGF
        Sbjct  61   FKDSLSINATNIKHFKNCTSISGDLHILPVAFRGDSFTHTPPLDPQELDILKTVKEITGF  120

        Query  121  LLIQAWPENRTDLHAFENLEIIRGRTKQHGQFSLAVVGLDITSLGLRSLKEISDGDVIVS  180
                     LLIQAWPENRTDLHAFENLEIIRGRTKQHGQFSLAVV L+ITSLGLRSLKEISDGDVI+S
        Sbjct  121  LLIQAWPENRTDLHAFENLEIIRGRTKQHGQFSLAVVSLNITSLGLRSLKEISDGDVIIS  180

        Query  181  GNQKLCYANTINWKKLFGTSSQKTKIINNKDEKGCKAIGHVCHPLCSSEGCWGPEPKDCV  240
                     GN+ LCYANTINWKKLFGTS QKTKII+N+ E  CKA G VCH LCS EGCWGPEP+DCV
        Sbjct  181  GNKNLCYANTINWKKLFGTSGQKTKIISNRGENSCKATGQVCHALCSPEGCWGPEPRDCV  240

        Query  241  SCQNVTRGKECVEKCNVLEGEPREFVENSECIQCHPECLPQAMNIT  286
                     SC+NV+RG+ECV+KCN+LEGEPREFVENSECIQCHPECLPQAMNIT
        Sbjct  241  SCRNVSRGRECVDKCNLLEGEPREFVENSECIQCHPECLPQAMNIT  286
```

Supplementary Figure 1. Alignment of a region of 286 a.a. encompassing the whole domain responsible for Cetuximab binding (domain III) in feline *vs* human EGFR protein. Aminoacidic sequences are from ALJ56200.1 (Feline EGFR, a.a. 189-474) and AIC61960.1 (Human EGFR, a.a. 240-525).

Supplementary Figure 2

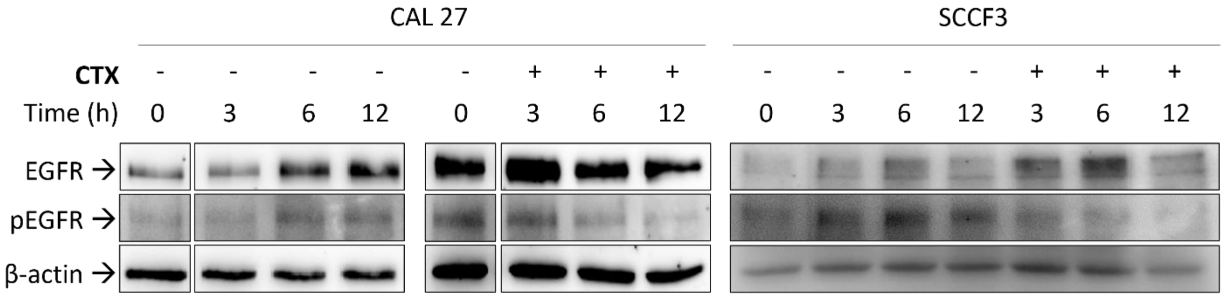

Supplementary Figure 2. Assessment of experimental conditions for evaluation of EGFR inhibition by Cetuximab. Cetuximab-sensitive CAL 27 and feline oral squamous cell carcinoma cell line SCCF3 were incubated (+) or not (-) with Cetuximab (CTX) at 100 µg/mL and harvested after 3, 6 and 12 hours (h) to be analysed by western blotting (WB) for EGFR and phospho-EGFR (pEGFR). The treatment induced a time-dependent reduction of pEGFR levels in both cell lines, with highest effect at 12 h. WB for β-actin antibody ensured comparable protein loading. Paired boxes in untreated (-) CAL 27 are cut from the same membrane at the same exposure time and properly aligned according to the molecular marker loaded onto the gel, as well as those in treated (+) cells.

Supplementary Figure 3

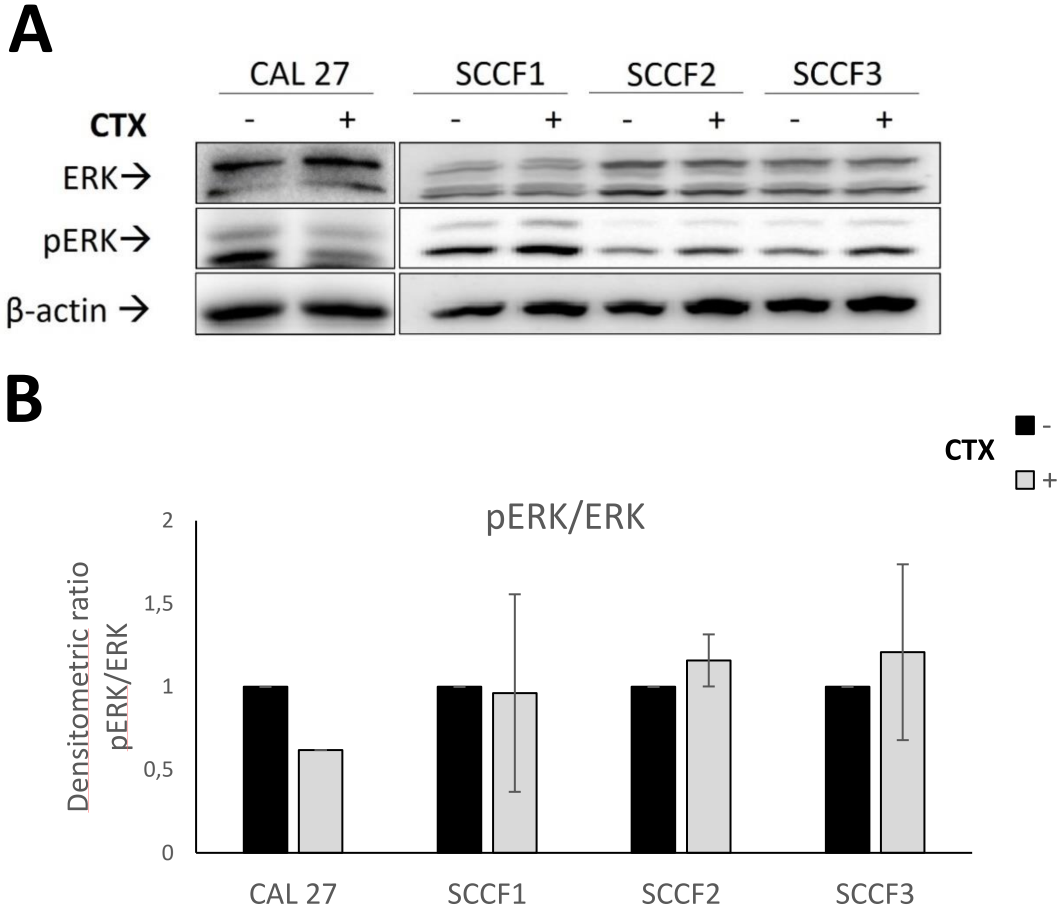

Supplementary Figure 3: Effects of Cetuximab on ERK pathway in feline oral squamous cell carcinoma cell lines (SCCF1, SCCF2, SCCF3) and CAL 27. **(A)** Cells were incubated with Cetuximab (CTX) at 100 µg/mL for 12 hours and analysed by western blotting (WB) for ERK and phospho-ERK (pERK). The treatment (+) induced the expected decrease in pERK compared to untreated control (-) in CAL 27 but not in feline cell lines. WB for β-actin ensured comparable protein loading and allowed normalization. **(B)** Densitometric analysis of pERK levels expressed as densitometric ratio pERK/ERK. Standard deviations are from two repeated, independent experiments.

Supplementary Figure 4

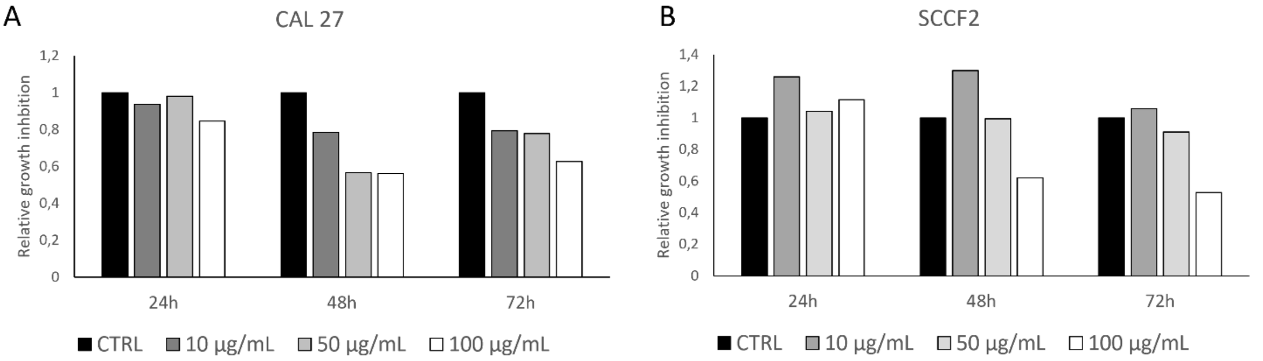

Supplementary Figure 4: Assessment of experimental conditions for evaluation of cell growth inhibition by Cetuximab. Cetuximab-sensitive CAL 27 **(A)** and feline oral squamous cell carcinoma cell line SCCF2 **(B)** were incubated with Cetuximab at 10, 50 and 100 µg/mL, harvested after 24, 48 and 72 hours (h), and relative growth inhibition with respect to untreated control (CTRL) was calculated as previously described (1). The treatment induced growth inhibition in both cell lines, at different doses and times of incubation.

1. Altamura G, Degli Uberti B, Galiero G, De Luca G, Power K, Licenziato L, et al. The Small Molecule Bibr1532 Exerts Potential Anti-Cancer Activities in Preclinical Models of Feline Oral Squamous Cell Carcinoma through Inhibition of Telomerase Activity and Down-Regulation of Tert. Front Vet Sci (2020) 7:620776. doi: 10.3389/fvets.2020.620776.

Supplementary Figure 5

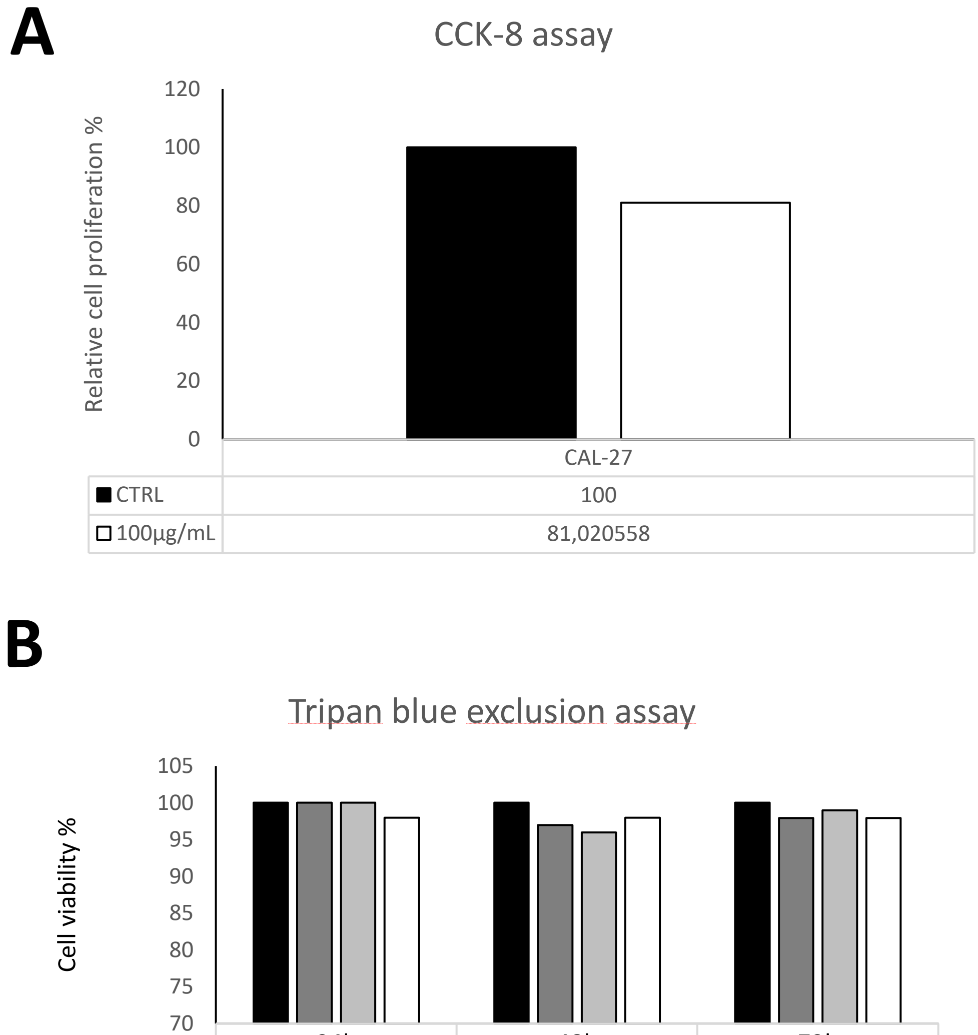

	CAL-27
■ CTRL	100
□ 100µg/mL	81,020558

	24h	48h	72h
■ CTRL	100	100	100
■ 10 µg/mL	100	96,96969697	97,93814433
■ 50 µg/mL	100	95,95959596	98,96907216
□ 100 µg/mL	97,95918367	97,97979798	97,93814433

Supplementary Figure 5. Impact of Cetuximab on cell proliferation and viability in CAL 27. (A) Cells treated with Cetuximab at 100 µg/mL were analyzed by CCK-8 assay after 48 hours (h). The treatment induced an impairment of cell proliferation. **(B)** Cells treated at 10, 50 and 100 µg/mL were analyzed by trypan blue exlclusion assay after 24, 48 and 72 h. The treatment induced a slight decrease in cell viability also at longer incubation times.

Supplementary Figure 6

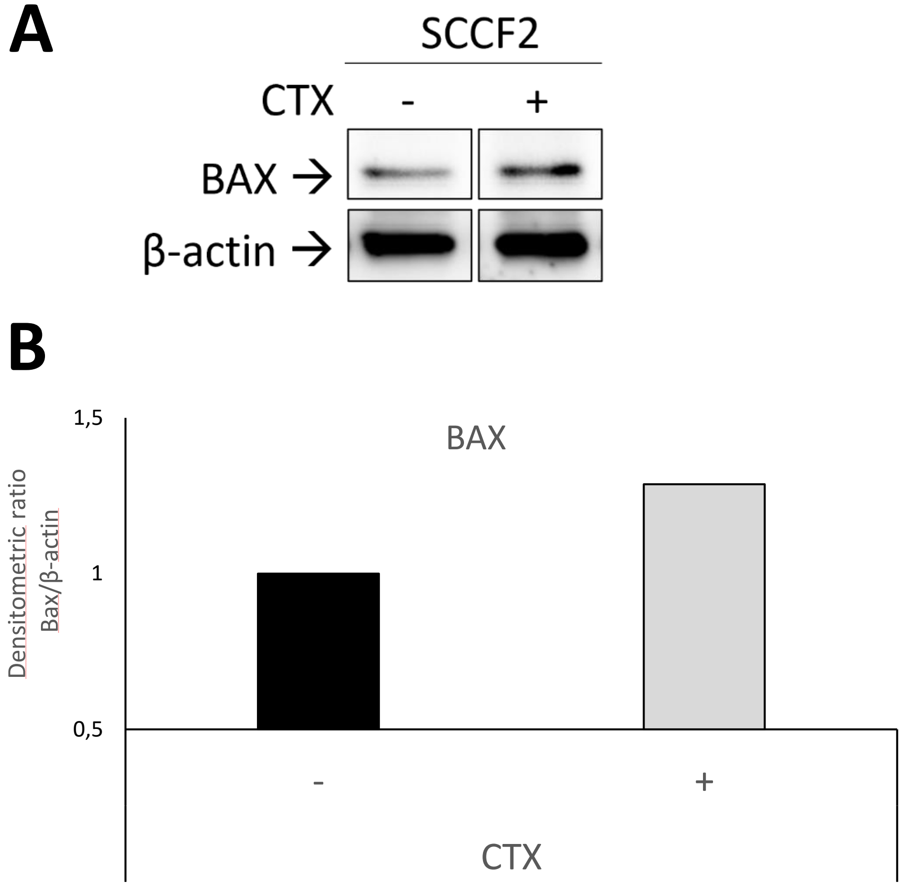

Supplementary Figure 6. Western blotting for the proapoptotic marker BAX in feline oral squamous cell carcinoma cell line SCCF2 treated with Cetuximab. (A) Cells treated with Cetuximab (CTX) at 100 µg/mL were analyzed by western blotting (WB) for BAX after 48 hours. The treatment (+) induced an increase of BAX compared to untreated control (-). WB for β-actin antibody ensured comparable protein loading and allowed normalization. Paired boxes for each cell line are cut from the same membrane at the same exposure time and properly aligned according to the molecular marker loaded onto the gel. Full scans from original gels are shown in Supplementary Figure 7 (see below). **(B)** Densitometric analysis of BAX expressed as densitometric ratio with β-actin.

Supplementary Figure 7

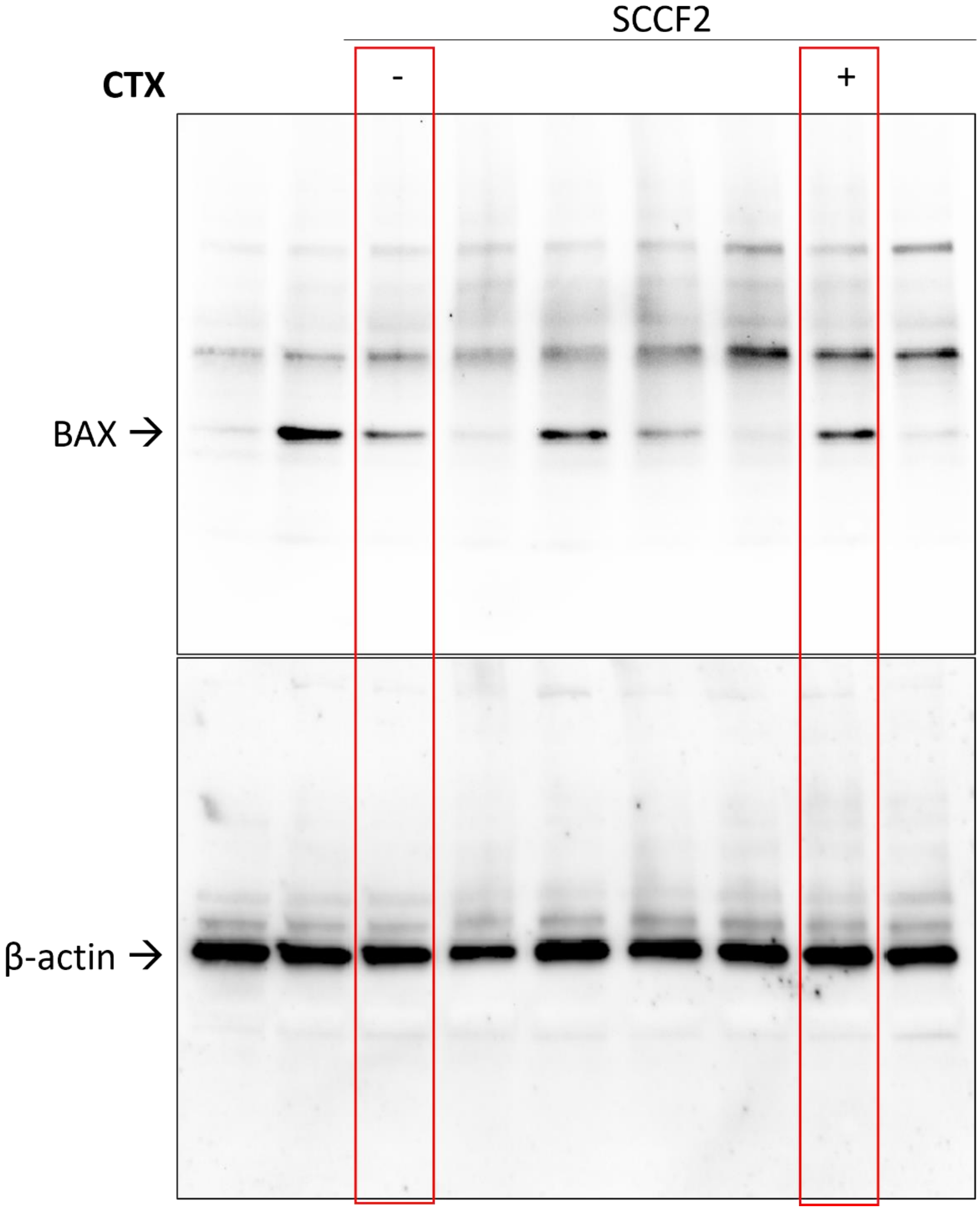

Supplementary Figure 7. Full scans from the entire original gels of Western blotting experiments for BAX shown in Supplementary Figure 6. Red rectangles indicate the samples included in the figure.

Supplementary Figure 8

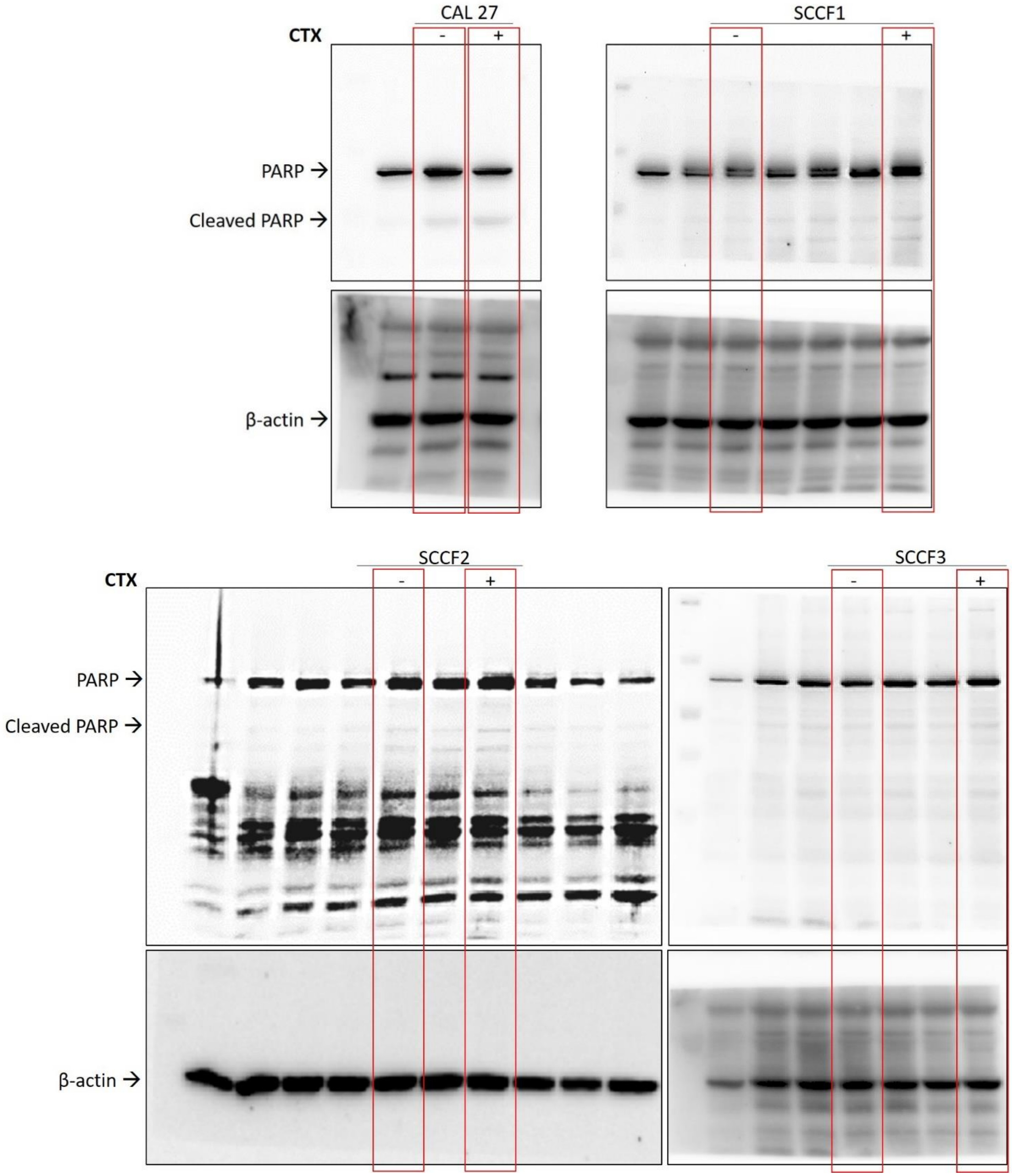

Supplementary Figure 8. Full scans from the entire original gels of Western blotting experiments for PARP shown in Figure 4. Red rectangles indicate the samples included in the figure.
